# Summary BSR Annual Meeting 2018

**DOI:** 10.5334/jbsr.1669

**Published:** 2018-11-17

**Authors:** Raymond Oyen

**Affiliations:** 1 UZ Leuven, BE

Dear Colleagues,

The Annual Meeting of the BSR edition 2018 focusses on head and neck radiology and interventional radiology. In addition and on the initiative of the Young Radiologist Section (YRS) a dedicated session will largely embrace the current hype of artificial intelligence.

As in previous years, the Sections have put a lot of efforts in realizing a scientific programme with a national and international faculty of experts.

After the scientific programme, you are kindly invited to a party-like social event to further enhance contacts in a relaxing environment!

We take this opportunity to draw your attention to the International Day of Radiology 2018 (November 8) with ‘Cardiac Imaging’ as the main theme.

It will be a great pleasure to welcome you at the 2018 Annual Meeting of the BSR in Brussels.

Best regards.

Prof. Dr. Raymond Oyen

On behalf of the Scientific Council of the BSR

November 2018

